# 

**Published:** 1896-09

**Authors:** 


*We positively cannot send premium to any subscriber who does not pay a year in advance.*

Notice.—Our offer of a thermometer to *all* cash-in-advance subscribers is withdrawn. *New* subscribers, paying full year in advance, may still get the thermometer. Book offer still open to all who pay a year in advance. "Stories of a Country Doctor."

### AMERICAN ASSOCIATION OF OBSTETRICIANS AND GYNECOLOGISTS.

The ninth annual meeting of the American Association of Obstetricians and Gynecologists will be held at the Hotel Jefferson, Richmond, Va., Tuesday, Wednesday, and Thursday, September 22, 23, and 24, 1896.

The proprietors of the "Jefferson" offer special rates to the fellows of the association, their families, and guests, as well as to any physicians who come to attend the meeting. It is confidently expected that the railways will offer transportation at a uniform rate of a fare and a third on the certificate plan to all in attendance. Let all obtain certificates from their local ticket agents, or from the nearest point where certificates are granted.

### OUTLINE PROGRAM.

The association will meet in executive session with closed doors on Tuesday, September 22d, at 9:30 o'clock A. M., for the election of new fellows. The open session for the reading of papers will begin at 10 o'clock A. M. Recess for luncheon 1 o'clock P. M. Afternoon session at 3 o'clock P. M. An evening session will be held Tuesday at 8 o'clock.

Morning session will begin Wednesday at 9:30 o'clock for the reading of scientific papers. Recess at 1 o'clock. Afternoon session at 3 o'clock. Adjournment at 5 o'clock. Executive session at 6:30 o'clock. Annual dinner at 8 o'clock P. M.

Tuesday morning the session will begin at 10 o'clock. Recess at 1 o'clock. Afternoon session at 3 o'clock. Final adjournment at 5 o'clock. A full attendance is specially requested at the final session.

### PAPERS PROMISED.

NOTE.—No attempt is made to arrange papers in the order in which they are to be read. That will be done in the permanent program.

1. Principles and Progress in Gynecology. President's address. Joseph Price, Philadelphia.

2. Vaginal Hysterectomy by the Clamp Method, Sherwood Dunn, Los Angeles.

3. Further Experience with Appendicitis, A. Vander Veer, Albany.

4. Relation of Malignant Disease of the Adnexa to Primary Invasion of the Uterus, A. P. Clarke, Cambridge.

5. Treatment of Puerperal Septicemia, H. W. Longyear, Detroit.

6. Treatment of Posterior Presentation of the Vertex, E. P. Bernardy, Philadelphia.

7. Relation of Local Visceral Disorders to the Delusions and Hallucinations of the Insane, W. P. Manton, Detroit.

8. Differential Diagnosis of Hemorrhage, Shock, and Sepsis, Eugene Boise, Grand Rapids.

9. Movable Kidney; Local and Remote Results, A. H. Cordier, Kansas City.

10. Pathology and Indications for Active Surgical Treatment in Contusions of the Abdomen, W. G. Macdonald, Albany.

11. Some Causes of Insanity in Women, George H. Rohé, Sykesville.

12. Subject to be announced, John Milton Duff, Pittsburg.

13. Shall Hysterectomy be Performed in Inflammatory Diseases of the Appendages? L. H. Dunning, Indianapolis.

14. Subject to be announced, Rufus B. Hall, Cincinnati.

15. Subject to be announced, George Ben Johnson, Richmond.

16. Dynamic Ileus, with Report of Cases, J. W. Long, Richmond.

17. Faradic Treatment of Uterine Inertia and Subinvolution, Charles Stover, Amsterdam.

18. A Plea for Absorbable Ligatures, H. E. Hayd, Buffalo.

19. Treatment of the Stump, J. F. Baldwin, Columbus.

20. Limitations in the Teaching of Obstetrics and Gynecology as Determined by State Medical Examining Boards, William Warren Potter, Buffalo.

21. Subject to be Announced, Walter B. Chase, Brooklyn.

22. (*a*) The Philosophy of Drainage; (*b*) Treatment of the Pedicle in Hysterectomy or Hystero-Myomectomy in the Abdominal Method, Geo. F. Hulbert, St. Louis.

23. Removal of the Uterine Appendages for Epilepsy and Insanity; a Plea for its More General Adoption, D. Tod Gillaim, Columbus.

24. Albuminuria of Pregnancy, A. Fr. Eklund, Stockholm.

25. Subject to be announced, Lawson Tait, Birmingham.

26. Unnecessary and Unnatural Fixation of the Uterus and its Results, James F. W. Ross, Toronto.

27. Sarcoma of the Urethra, Charles A. L. Reed, Cincinnati.

28. Appendicitis as a Complication in Suppurative Inflammation of the Uterine Appendages, L. S. McMurtry, Louisville.

29. Gunshot Wounds of the Abdomen with the New Gun, J. D. Griffith, Kansas City.

30. Subject to be announced, Walter B. Dorsett, St. Louis.

31. Subject to be announced, W. E. B. Davis, Birmingham.

32. Subject to be announced, E. Arnold Praeger, Los Angeles.

33. Tubo-Ovarian Cysts with Interesting Cases, A. Goldspohn, Chicago.

34. Obstruction of the Bowels Following Abdominal Section, Geo. S. Peck, Youngstown.

35. Memorial of Dr. Hiram Corson, Traill Green, Easton.

Correspondence is pending concerning additional papers. All titles must be offered before August 25th, when the permanent program goes to press. The executive council directs attention to the following by-law:

PAPERS.

VI. The titles of all papers to be read at any annual meeting shall be furnished to the secretary *not later* than one month before the first day of the meeting.

No paper shall be read before the association that has already been published or that has been read before any other body.

Not more than thirty minutes shall be occupied in reading any paper before the association.

Abstracts of all papers read should be furnished to the secretary at the meeting.

All papers read before the association shall become its sole property if accepted for publication; and the executive council may decline to publish any paper *not handed to the secretary complete* before the final adjournment of the annual meeting.

Dr. George Ben Johnson, 407 E. Grace street, Richmond, Va., is chairman of the committee of arrangements, who should be addressed in regard to hotel accommodations and railway fares.

JOSEPH PRICE, President.

WILLIAM WARREN POTTER, Secretary.

---

CÆSAREAN SECTION.—Readers will be interested in the perusal of Dr. Allred's description (see p. 452) of his operation for Cæsarean Section; and whether or not they may concur in the choice of such procedure under the circumstances, they will certainly be inclined to heartily congratulate the patient and him on the successful issue of what must be looked upon as one of the most extraordinary abdominal sections on record.

---

### ABOUT COCAINE.

Following is a summary of an interesting paper on the "Rational Use of Cocaine in Surgery." (Dr. C. A. Dundore, *Medical and Surgical Reporter*):

1. The use of cocaine in surgery should not be abandoned because its irrational employment has produced deleterious results.
2. Always make a thorough physical examination of the patient before injecting the drug.
3. It should not be used in cases showing organic disease of the brain, heart, lungs, or kidneys, or in persons of a neurotic diathesis.
4. Children bear it fully as well as adults.
5. The patient should always be placed in a recumbent position prior to its employment.
6. Constriction should be used, whenever possible, to limit the action of the drug to the desired area.
7. Use a freshly prepared solution for each case.
8. Distilled water is to be employed, to which phenic, salicylic, or boric acid is to be added.

